# *Colletotrichum siamense*, a Novel Causal Agent of *Viburnum odoratissimum* Leaf Blotch and Its Sensitivity to Fungicides

**DOI:** 10.3390/jof9090882

**Published:** 2023-08-28

**Authors:** Hui Li, Yang-Chun-Zi Liao, Yu Wan, De-Wei Li, Li-Hua Zhu

**Affiliations:** 1 College of Forestry, Nanjing Forestry University, Nanjing 210037, China; lhui@njfu.edu.cn (H.L.); yangchunziliao@njfu.edu.cn (Y.-C.-Z.L.); wanyu@njfu.edu.cn (Y.W.); 2 Co-Innovation Center for Sustainable Forestry in Southern China, Nanjing Forestry University, Nanjing 210037, China; 3 The Connecticut Agricultural Experiment Station Valley Laboratory, Windsor, CT 06095, USA

**Keywords:** *Viburnum odoratissimum*, new disease, *Colletotrichum siamense*, fungicides, phylogeny

## Abstract

*Viburnum odoratissimum* Ker-Gawl is native to Asia and is usually used as a garden ornamental. In September 2022, a leaf blotch on *V. odoratissimum* was observed in Nanjing, Jiangsu, China. The disease causes the leaves of the plants to curl and dry up and defoliate early. It not only seriously affects the growth of the plants but also greatly reduces the ornamental value. The pathogenic fungus was isolated from the diseased leaves, and the fungus was identified to be *Colletotrichum siamense* based on morphological features and multilocus phylogenetic analyses of the internal transcribed spacer (ITS) region, actin (*ACT*), calmodulin (*CAL*), beta-tubulin 2 (*TUB2*), chitin synthase (*CHS-1*), Apn2-Mat1-2 intergenic spacer and partial mating type (*ApMat*), and glyceraldehyde-3-phosphate dehydrogenase (*GAPDH*) genes. Pathogenicity tests were performed by inoculating healthy leaves with conidia. *C. siamense* can grow at 15–35 °C, with an optimal growth temperature at 25–30 °C. The results of sensitivity to nine fungicides showed that *C. siamense* was the most sensitive to prochloraz in the concentration range of 0.01 μg/mL to 100 μg/mL. Therefore, spraying prochloraz before the optimum growth temperature of pathogenic fungus can achieve effective control. It provided useful information for future studies on the prevention and treatment strategies of *C. siamense*. This is the first report of leaf blotch caused by *C. siamense* on *V. odoratissimum* in China and worldwide.

## 1. Introduction

*Viburnum odoratissimum* Ker-Gawl (Adoxaceae) is native to Asia [1]. It is widely cultivated as an ornamental and medicinal plant and mainly cultivated along roadsides in urban landscapes and in parks as well as residential areas [2].

In recent years, large-scale cultivation has increased occurrence of diseases, which affected the growth of plants and reduced the ornamental value. For example, anthracnose caused by *C. gloeosporioides* in China [2], powdery mildew caused by *Erysiphe hedwigii* [3,4], leaf spots caused by *Alternaria* spp. [5], *Diaporthe eres* [6], *Neofusicoccum parvum* [7], and *Corynespora cassiicola* [8] have been reported in China. These fungal diseases have variable effects on *V. odoratissimum* growth and industrial development [2], and more studies are necessary. The host–pathogen relationship of *V. odoratissimum* needs further studies, and more pathogens may be discovered.

*Colletotrichum* is usually reported as phytopathogens, epiphytes, saprobes, or endophytes, and it is one of the most important phytopathogens in the world [9,10,11,12,13,14]. *Ca*.16 species complexes of the genus *Colletotrichum* have been described worldwide [15]. Some species are also pathogenic to humans and animals. For example, *C. gloeosporioides* sensu lato and *C. dematium* were reported as the cause of fungal keratitis [16,17]. *C. acutatum* have been reported to infect scale insects [18]. As plant pathogens, *Colletotrichum* infects many species of forest trees, grasses, economic fruits, and crops, resulting in great economic losses [19,20,21,22,23,24]. In addition, it is common for multiple *Colletotrichum* species to infect a single host species [25].

The early taxonomic system for *Colletotrichum* based on morphology and host–plant association was deficient and to a great extent hindered research on species diversity [26,27,28,29,30]. It is of great significance to improve the accuracy of plant pathogen identification for effective control of plant diseases [26,31]. Cai et al. [26] proposed a polyphasic method based on morphology and multi-locus phylogeny to identify *Colletotrichum* species, which significantly advanced the taxonomy of the *Colletotrichum* species [32,33,34]. For example, a combination of multiple locus sequences, including the internal transcribed spacer (ITS) region, actin (*ACT*), calmodulin (*CAL*), beta-tubulin 2 (*TUB2*), chitin synthase (*CHS-1*), and glyceraldehyde-3-phosphate dehydrogenase (*GAPDH*) genes/region, can provide additional molecular features for more accurate identification of *Colletotrichum* species [35,36].

At present, the prevention and control of anthracnose rely mainly on chemical control. For example, Gao et al. [37] tested five different fungicides to control pepper anthracnose and found that mefenfluconazole had the best curative effects. Kim et al. [38] tested four fungicides for sensitivity analysis on 25 different *Colletotrichum* species, and the results showed that they were highly sensitive to tebuconazole. Mora-Aguilera et al. [39] tested benomyl, carbendazim, and prochloraz for sensitivity analysis on five different isolates of the *Colletotrichum gloeosporioides* species complex from mango orchards in Mexico. He et al. [40] found that five *Colletotrichum* species from different hosts were highly sensitive to six fungicides, including pyraclostrobin, difenoconazole, fludioxonil, tebuconazole, pyrisoxazole, and tetramycin. In addition, different *Colletotrichum* species have different sensitivities to the same fungicide [41].

Considering the geographical and host differences of *Colletotrichum* spp., appropriate fungicides should be selected. Understanding the sensitivity of the isolates associated with *V. odoratissimum* leaf blight will make effective strategies for managing the disease. The objectives of this study were (i) to isolate and identify the pathogen of leaf blotch on *V. odoratissimum* based on morphology and multilocus phylogenetic analyses in Nanjing, Jiangsu, China (ii) to determine the pathogenicity of the fungus on *V. odoratissimum* and (iii) to study the inhibitory effects of nine fungicides on the pathogen by phenotyping experiments on media plates.

## 2. Materials and Methods

### 2.1. Sampling and Isolation

Diseased leaves were collected in September 2022 from Nanjing Forestry University, Jiangsu, China. The entire planting area was about 400 m^2^, and samples were taken within 20 m^2^. Twenty symptomatic leaves were collected from five infected 3-year-old plants and washed with distilled water. The leaf pieces (3 × 3 mm) at the intermediate area of diseased and healthy portions were cut off, sterilized in 75% ethanol solution for 30 s and in 1% NaClO for 90 s, rinsed three times in sterile water, dried with sterilized filter paper, and plated on potato dextrose agar (PDA) Petri dishes and incubated at 25 °C in the dark for 3 days [42]. Pure cultures were obtained by cutting hyphal tips, which were transferred to fresh PDA plates.

### 2.2. DNA Extraction, PCR Amplification and Sequencing

Fungal genomic DNA was extracted from aerial hyphae of 3-day-old cultures using the cetyltrimethylammonium bromide (CTAB) method [43]. Polymerase chain reaction (PCR) was used to amplify seven loci: ITS, *ACT*, *CAL*, *TUB2*, *CHS-1*, *ApMat*, and *GAPDH*, which were amplified with the primer pairs ITS1/ITS4 [44], ACT-512F/ACT-783R [45], CL1C/CL2C [36], T1/Bt2b [46,47], CHS-79F/CHS-345R [45], AM-F/AM-R [48], and GDF/GDR [49], respectively. The reaction conditions are shown in Table 1. The PCR products were purified and sequenced at the Sangon Biotech (Shanghai) Co., Ltd. (Nanjing, China).

### 2.3. Sequence Alignment and Phylogenetic Analyses

The resulting sequences were compared in GenBank using Blast, and the appropriate reference sequence and an outgroup were selected for phylogenetic analyses. The sequences were aligned and manually adjusted using MAFFT ver. 7.313 [50] and BioEdit ver. 7.0.9.0 [51], respectively. Hosts, locality, and GenBank accession numbers of the *Colletotrichum* species used for phylogenetic analyses in this study are presented in Table S1. Multilocus tandem data (ITS, *ACT*, *CAL*, *TUB2*, *CHS-1*, *ApMat*, and *GAPDH*) were used for phylogenetic analyses using PhyloSuite v1.2.1 [52]. Maximum likelihood (ML) and Bayesian inference (BI) analyses were performed using IQtree ver. 1.6.8 [53] and MrBayes ver. 3.2.6 [54], respectively. Phylogenetic trees were drawn with FigTree ver. 1.4.4 (http://tree.bio.ed.ac.uk/software/figtree/ accessed on 1 April 2023).

### 2.4. Morphological Characteristics and Biological Characteristics

The isolates were plated in the center of potato dextrose agar medium (PDA), Czapek Dox agar (CZA), minimal methanol medium (MM), and complete medium (CM) and incubated at 25 °C with a 12/12 h light/dark cycle. Colony characteristics of the cultures were observed after 3 days. Acervuli and conidial masses were observed under a Zeiss stereo microscope (SteRo Discovery v20, Oberkochen, Germany). The morphological characteristics, such as shape, color, septation, and size of 30 conidia, conidiophores, acervuli, appressoria, and setae, were observed and measured under a Zeiss Axio Imager A2m microscope (Carl Zeiss, Oberkochen, Germany), respectively. Appressoria of the isolate were induced from conidia using a slide culture technique [26,55]. 

To determine the optimal growth temperature for the isolates, mycelial plugs (5 mm diam.) were placed on fresh PDA and incubated at 15, 20, 25, 30, and 35 °C, and colony diameters were recorded daily. Experiments were conducted at five temperatures, with each isolate having three replicates. The experiment was conducted two times.

### 2.5. Pathogenicity Tests

To verify the Koch’s postulates, three representative isolates (SHS 1-3, SHS 1-6, and SHS 1-7) and nine 3-year-old healthy *V. odoratissimum* seedlings were used for inoculation in pathogenicity test. The tested plants were taken from Xinrun Green Seedling Base in Nanjing, Jiangsu Province, China. Healthy leaves were wounded with a sterile needle, and 10 µL conidial suspensions (10^6^ conidia/mL) of the isolates were inoculated, respectively. Five leaves were inoculated with each isolate. Healthy leaves from three additional seedlings treated with sterilized H_2_O were used as the control group. Conidia were produced in PDA plates incubated at 25 °C for 7–10 days. Conidial suspensions were prepared with sterile water, and the concentration was adjusted using a haemocytometer. The seedlings with inoculated leaves and control seedlings were placed in a tent (1.5 × 1.2 × 1.5 m) with a humidifier (300 mL/h) to maintain RH 70%. The tent was placed in a greenhouse at 25 ± 2 °C and observed daily for one week. All experiments were conducted three times.

### 2.6. Susceptibility of Colletotrichum Isolates to Fungicides

The fungicides were provided by the laboratory of Nanjing Agricultural University: prochloraz [96% active ingredient (a.i); Jiangshan Pesticide Chemical Co., Ltd., Nantong, China], tebuconazole [97% a.i; Fengdeng Pesticide Co., Ltd. Changzhou, China], myclobutanil [97% a.i; United Pesticide Industry Co., Ltd., Taian, China.], and pyraclostrobin [98% a.i; Kangqiao Biological Co., Ltd., Jinan, China], carbendazim [97% a.i; Lanfeng Biochemical Co., Ltd., Xuzhou, China], flusilazole [95% a.i; Bailingwei Technology Co., Ltd., Beijing, China], difenoconazole [96% a.i; Syngenta Crop Protection Co., Ltd., Nantong, China], imazalil [97% a.i; Tesco Chemical Co., Ltd., Wuhan, China], and iprodione [96% a.i; Yifan Biotechnology Co., Ltd., Wenzhou, China] were used in this study. Mycelial plugs (5 mm diam.) were removed from the edge of colonies of 5-day-old cultures actively growing on PDA and placed at the center of PDA plates with fungicide at different dosages or without fungicide (as a control). For the nine fungicides, final concentrations of 0.01, 0.1, 1, 10, and 100 μg/mL were added to the amended media. The plates were incubated for 3 days at 25 °C, and the diameter of each colony was measured in perpendicular directions. Each isolate and concentration had three replicates, respectively. All experiments were conducted two times. The formula to calculate the inhibition rate (%) is [1 − (colony diameter at fungicide concentration/colony diameter of the control)] × 100. EC50 values (the concentration inhibitory growth for 50%) were estimated by regression to the log_10_ probability conversion of percentage inhibition to fungicide concentration.

## 3. Results

### 3.1. Disease Symptoms and Fungal Isolation

**Figure 1:**
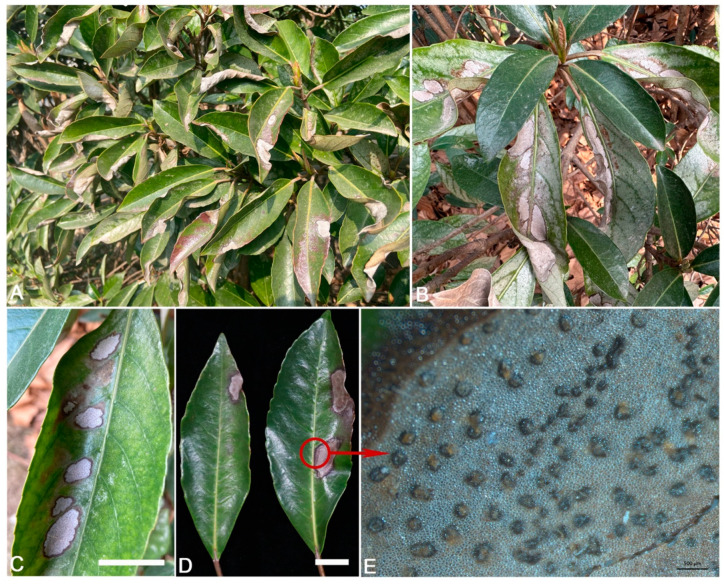
Symptoms of leaf blotch on *Viburnum odoratissimum* in the field. (**A**–**D**). Naturally infected leaves, scale bar = 1 cm. (**E**). Acervuli and conidial masses, scale bar = 500 μm.

From September to December 2022, anthracnose leaf blotch was observed on *V. odoratissimum* with 54% (108/200 plants) of disease incidence in Nanjing, Jiangsu, China. On average, 60% of the leaves on each *V. odoratissimum* plant were infected by this disease. Symptoms began as small lesions (5–10 mm in diameter) with brown necrotic centers, which gradually expanded and merged to irregular large necrotic spots; the leaves curled inward when the infected area expanded to ca. ¼ of a leaf blade and defoliated (Figure 1A–C). The centers of the spots were gray and surrounded by dark brown edges (Figure 1D). Numerous acervuli developed on the spots (Figure 1E). In total, 80 fungal isolates were isolated from diseased leaves of *V. odoratissimum*. Two types of colonies were found according to ITS sequences and the colony morphology, and one was consistently associated with the disease with an average isolation frequency of 95% (76/80). The ten representative isolates isolated from diseased host (SHS 1-1, SHS 1-2, SHS 1-3, SHS 1-4, SHS 1-5, SHS 1-6, SHS 1-7, SHS 1-8, SHS 1-9, and SHS 1-10) were chosen for further study.

### 3.2. Phylogenetic Analyses

**Figure 2:**
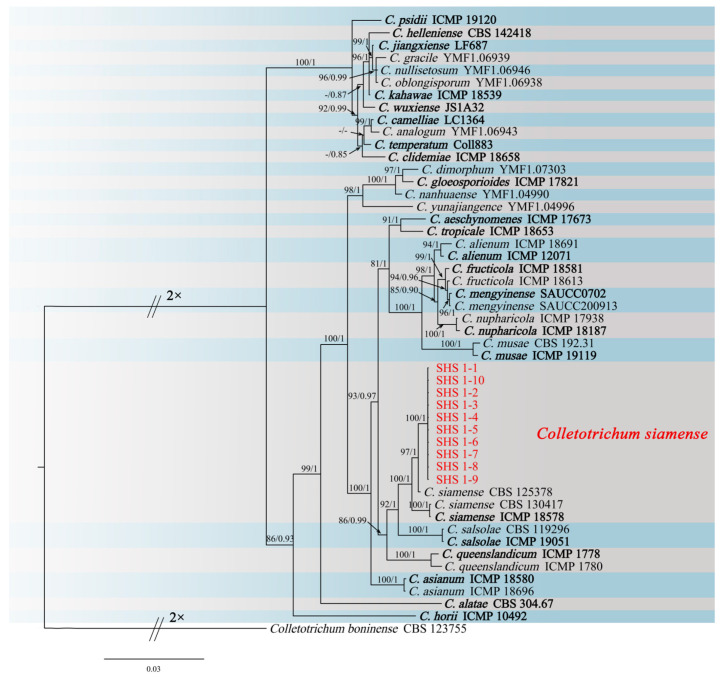
Phylogenetic relationship of SHS 1-1, SHS 1-2, SHS 1-3, SHS 1-4, SHS 1-5, SHS 1-6, SHS 1-7, SHS 1-8, SHS 1-9, and SHS 1-10 with related taxa derived from the concatenated sequences of ITS, *ACT*, *CAL*, *CHS-1*, *TUB2*, *ApMat*, and *GAPDH* genes/region using the maximum likelihood and Bayesian posterior probability analyses. RAxML bootstrap support values (ML ≥ 80) and Bayesian posterior probability values (PP ≥ 0.80) were shown at the nodes (ML/PP). *Colletotrichum boninense* CBS 123755 is used as an outgroup. Bar = 0.03 substitution per nucleotide position. The sequences from this study are in red. The ex-type strains are in bold.

The genes/region ITS, *ACT*, *CAL*, *TUB2*, *CHS-1*, *ApMat*, and *GAPDH* from the 10 isolates (SHS 1-1, SHS 1-2, SHS 1-3, SHS 1-4, SHS 1-5, SHS 1-6, SHS 1-7, SHS 1-8, SHS 1-9, and SHS 1-10) were deposited in GenBank. Phylogenetic analyses using IQtree v.1.6.8 and MrBayes v.3.2.6 were based on the consistent analysis of the concatenated sequences of seven loci of the 10 isolates and 40 related sequences of *Colletotrichum* species from NCBI (Table S1). In the maximum likelihood phylogenetic tree, the ten isolates and *C. siamense* (CBS 125378) were in the same clade and had 97% RAxML bootstrap support (Figure 2). The phylogenetic tree obtained by Bayesian inference analysis was consistent with the maximum likelihood tree. In Bayesian inference analysis, the isolates were clustered in the same clade of *C. siamense* (CBS 125378) with a posterior probability value of 1. Based on the morphology and phylogeny, the fungus was identified as *C. siamense* sensu stricto.

### 3.3. Morphological Identification and Biological Characteristics

*Colletotrichum siamense* Prihast., L. Cai and K.D. Hyde.

**Figure 3:**
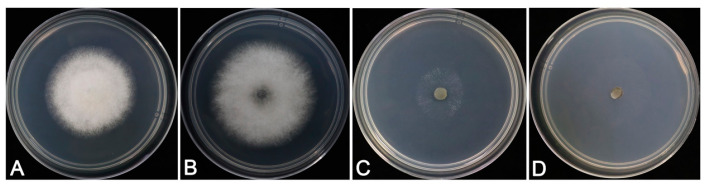
Colony characteristics of representative isolates SHS 1-7 isolated from *Viburnum odoratissimum* cultured after 3 days on CM, CZA, MM, and PDA media at 25 °C. (**A**). CM; (**B**). PDA; (**C**). MM; (**D**). CZA.

**Figure 4:**
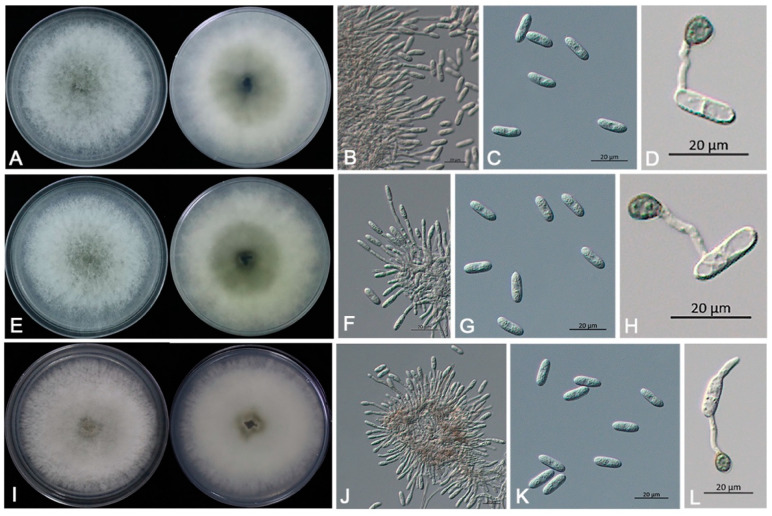
Morphological characteristics of *Colletotrichum siamense* representative isolates SHS 1-3, SHS 1-6, and SHS 1-7. (**A**–**D**). Isolate SHS 1-3: (**A**). fungal colony on PDA, 5 d growth from above and below, (**B**). conidiophores and conidia, (**C**). conidia, (**D**). conidium and appressorium. Scale bars: (**A**–**D**) = 20 μm. (**E**–**H**). Isolate SHS 1-6: (**E**). fungal colony on PDA, 5 d growth from above and below, (**F**). conidiophores and conidia, (**G**). conidia, (**H**). conidium and appressorium. Scale bars: (**E**–**H**) = 20 μm. (**I**–**L**). Isolate SHS 1-7: (**I**). fungal colony on PDA, 5 d growth from above and below. (**J**). conidiophores and conidia, (**K**). conidia, (**L**). conidium and appressorium. Scale bars: (**I**–**L**) = 20 μm.

The characteristics of colonies varied among the types of media. Colonies on CM with aerial mycelium dense, cottony, white (Figure 3A) grew better on PDA medium. Fungal colonies on PDA were white to pale greenish-gray with dense and fast-growing aerial mycelia (Figure 3B), and the reverse was gray-green at the center (Figure 4A,E,I). The isolates showed few aerial hyphae on MM and CZA medium, and the growth of the isolate was weak (Figure 3C, D). The colonies showed slowest growth rates on MM (Table 2).

Acervuli were (79.1–) 94.3–141.3 (–156.5) × (78.3–) 90.6–114.9 (–127.2) µm (mean ± SD = 105.5 ± 15.2 × 100.1 ± 12.3 µm, *n* = 30), light brown to dark brown, circular to irregular (Figure 1E). Conidiophores were hyaline, smooth, branched, septate, cylindrical to clavate (Figure 4B, F, J), (11.8–) 17.8–31.4 (–37.4) × (2.3–) 2.7–4.0 (–4.4) µm (mean ± SD = 20.1 ± 6.0 × 3.0 ± 0.4 µm, *n* = 30). Conidia were one-celled, cylindrical, hyaline, rounded at both ends (Figure 4C, G, K), (14.4–) 15.5–17.4 (–18.5) × (5.2–) 5.5–6.3 (–6.6) µm (mean ± SD = 16.2 ± 1.1 × 5.8 ± 0.3 µm, *n* = 30). Setae were observed in the acervuli, (71.2–) 88.6–131.9 (–149.3) × (2.2–) 2.7–3.9 (–4.3) µm (mean ± SD = 104.4 ± 17.4 × 3.1 ± 0.5 µm, *n* = 30), pale brown to dark brown, smooth, 4–6 septate, straight or curved, with acute tips. Appressoria were (7.3–) 7.9–9.8 (–10.4) × (5.5–) 6.0–7.6 (–8.1) µm (mean ± SD = 8.2 ± 0.6 × 6.7 ± 0.5 µm, *n* = 30), solitary, light brown to dark brown, nearly spherical or ellipsoidal, smooth and thick-walled (Figure 4D,H,L). Morphological characteristics of the ten isolates were similar to *Colletotrichum gloeosporioides* species complex [36]. Based on both morphological study and phylogenetic analyses, the ten isolates of this pathogenic fungus were identified to be *C. siamense* in the *C. gloeosporioides* species complex.

**Figure 5:**
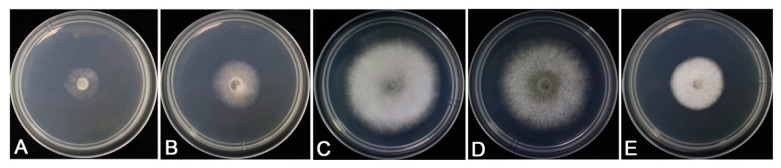
Colony morphology of the representative isolates SHS 1-7 growing on PDA medium for 3 days at 15, 20, 25, 30, and 35 °C. (**A**). 15 °C; (**B**). 20 °C; (**C**). 25 °C; (**D**). 30 °C; (**E**). 35 °C.

The isolates of *C. siamense* were grown in the range of 15–35 °C (Figure 5), and the colony hyphae grew the fastest, densest, and fluffiest in the range of 25–30 °C (Table 3). Thus, 25–30 °C was the optimal temperature for the growth of the isolates in the study. 

### 3.4. Pathogenicity of Fungal Isolates

**Figure 6:**
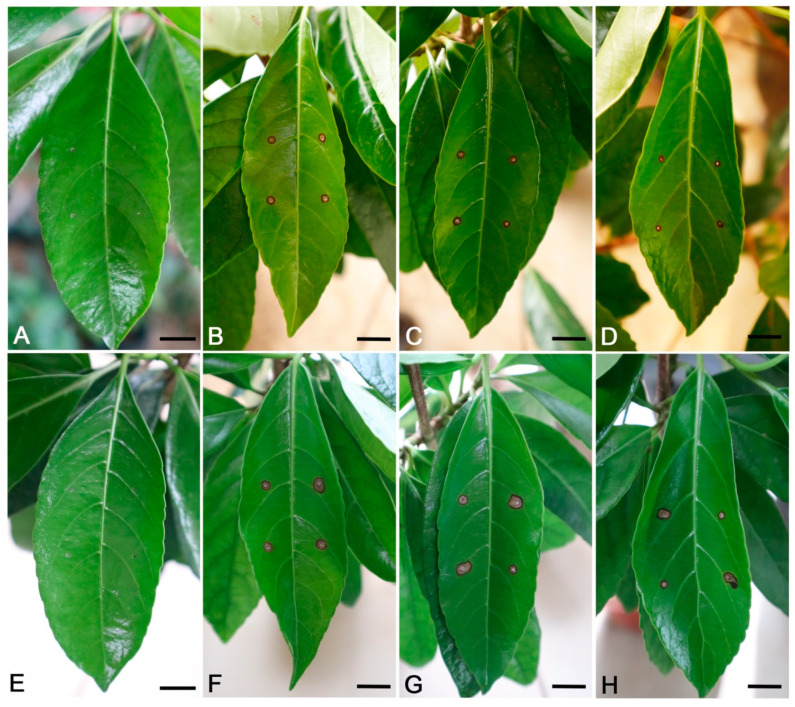
Pathogenicity of representative isolates of *Colletotrichum siamense* (SHS 1-3, SHS 1-6, and SHS 1-7) on *Viburnum odoratissimum*. (**A**). No symptoms observed on control leaf treated with sterile water after 3 days. (**B**–**D**). Symptoms on leaf inoculated with conidial suspensions of SHS 1-3, SHS 1-6, and SHS 1-7 after 3 days, respectively. (**E**). No symptoms observed on control leaf treated with sterile water after 7 days. (**F**–**H**). Symptoms on leaf inoculated with conidial suspensions of SHS 1-3, SHS 1-6, and SHS 1-7 after 7 days. Scale bars = 1 cm.

The three isolates (SHS 1-3, SHS 1-6, and SHS 1-7) were proven pathogenic to *V. odoratissimum* leaves. To complete Koch’s postulates, pathogenicity was tested on 3-year-old *V. odoratissimum* seedlings in the tent (moisture chamber). Three days post-inoculation, brown lesions were observed on leaves (Figure 6B–D). The lesions expanded after seven days (Figure 6F–H), whereas control plants were asymptomatic (Figure 6A,E). Although different isolates showed different virulence, they belonged to the same species in this study, and the difference in lesion diameter was small (Table 4). *C. siamense* was re-isolated from 100% of the inoculated leaves but not from the control, and *C. siamense* was confirmed based on morphological characters and the ITS sequences. Thus, *C. siamense* was the pathogen causing leaf blotch on *V. odoratissimum*. In the future work, unwounded inoculation will be conducted to further fulfill Koch’s postulates experiment.

### 3.5. Susceptibility of Colletotrichum Isolates to Fungicides

**Figure 7:**
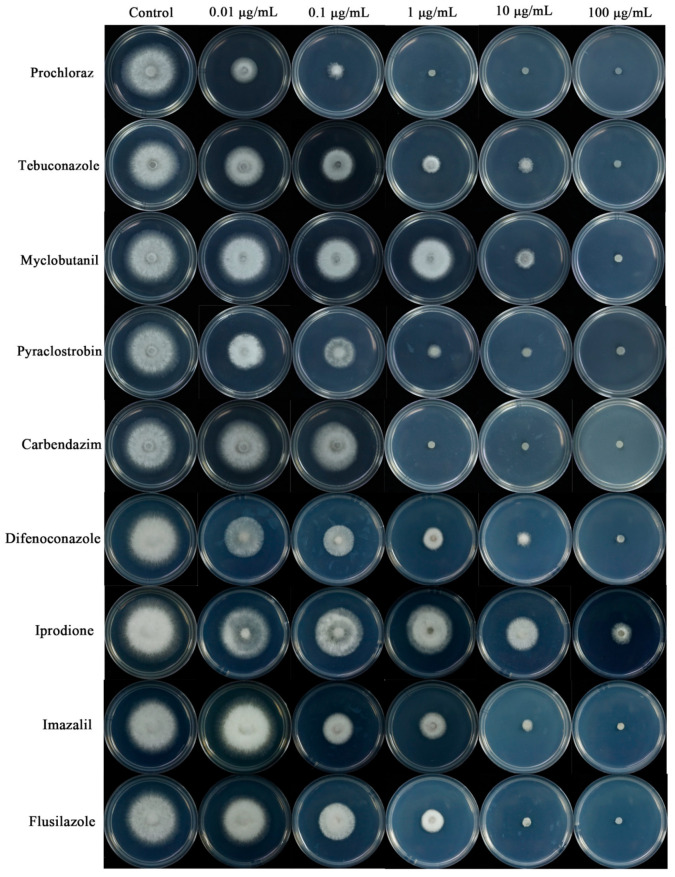
The effect of suppression on representative isolates SHS 1-7 on PDA plates at different doses of nine different fungicides (carbendazim, flusilazole, difenoconazole, imazalil, iprodione, myclobutanil, prochloraz, pyraclostrobin, and tebuconazole) based on fresh PDA medium assay for 3 days.

The nine fungicides all significantly inhibited the growth of the representative isolates on PDA, but the inhibitory effect of representative isolates caused by different fungicides were different. The inhibitory effect of prochloraz on the mycelial growth of representative isolates was the strongest (Table 5). The EC50 value of iprodione was the highest, but its inhibition on mycelium growth was the weakest. The experiments showed that prochloraz was the most effective fungicides at the same concentration against *V. odoratissimum* anthracnose in this study (Figure 7).

## 4. Discussion

*Colletotrichum* spp. have been reported in China, and most of them belong to the *C. gloeosporioides* species complex [56]. *Colletotrichum* species are important phytopathogens among many important economic hosts in the world. It has a wide distribution and numerous hosts and can infect the branches, leaves, and stems of *Citrus*, *Pyrus*, and *Camellia*, resulting in large-scale economic losses or poor plant growth [57,58,59]. *C. siamense*, a member of the *C. gloeosporioides* species complex, was first discovered on coffee in Thailand by Prihastuti et al. in 2009 [60]. *C. siamense* has been identified as part of the *gloeosporioides* species complex that can infect forest trees, fruits, and crops [36,61]. At present, reports of anthracnose are still increasing, and it is more important to effectively identify and distinguish *Colletotrichum* species. It is obviously difficult to accurately identify *Colletotrichum* species by only ITS sequence or morphology [26,62]. The polyphasic method combining morphology and multi-locus phylogenetic analyses, as well as pathogenicity tests to classify this genus has been recognized by researchers [26,35,36]. Therefore, the pathogen of leaf blotch was identified based on the combination of morphology and multi-locus phylogeny, which greatly improves the identification accuracy. With the development of molecular biology, more attention should be paid to studying the fungal diversity of host plants and how to counter the impact on host plants.

Temperature is one of the main factors affecting the prevalence of plant diseases and plays an important role in the infection process of anthracnose. Our study indicated that the optimum growth temperature of *C. siamense* ranged from 25 to 30 °C, which was basically consistent with other research reports [40,63]. Anthracnose may more frequently occur as pathogens under optimum growth temperature, so fungicides should be applied before the optimal temperature of the pathogen is reached [40,64]. 

The study on the sensitivity to fungicides showed that prochloraz had the best inhibitory effect on the growth of *C. siamense* at the same concentration, and the EC50 value was the lowest. These results are similar to those of Mora-Aguilera et al. [39], who reported that most of the isolates of *Colletotrichum* spp. from mango isolates were highly sensitive to prochloraz. Li et al. [65] detected that four *Colletotrichum* spp. of *Camellia oleifera* (oil-tea) anthracnose in the nursery were sensitive to prochloraz and indicated that the fungicide had not developed resistance to prochloraz at present. After testing 23 fungicides, Zhang et al. [66] found prochloraz to be the most toxic against mycelial growth of eight isolates of *C. gloeosporioides* in China. These results prove that prochloraz can be used as one of the effective fungicides to control anthracnose of *V. odoratissimum*, and it is also very valuable to prevent and control other plant diseases caused by *Colletotrichum* species. In addition, compared to other fungicides, pyraclostrobine, carbendazim, and difenoconazole had lower average EC50 values. These fungicides can also be used for preventing and treating anthracnose of *V. odoratissimum*. Notably, carbendazim and prochloraz are being reduced or banned in the market in several parts of the world, such as Australia, Canada, USA, and most European Union countries, due to their unfavorable toxicological properties [67,68]. Since these fungicides are still allowed to be produced and used in China, it is necessary to study new substitutes with less toxicities to human and environments.

## 5. Conclusions

In this study, *C. siamense* was identified as the pathogen of leaf blotch of *V. odoratissimum* based on pathogenicity tests, morphology, and multi-locus phylogenetic analyses. This finding also provides further evidence of the diversity of hosts for *C. siamense*, and multiple *Colletotrichum* species can infect the same host. The toxicity of nine fungicides to the pathogen was determined by phenotypic experiments on medium plates. These sensitivity data can be used as a reference for the change of the sensitivity of *Colletotrichum* spp. to different fungicides in the future. This is the first report of leaf blotch caused by *C. siamense* on *V. odoratissimum* in China and worldwide. This finding will provide useful information for future studies on the prevention and treatment strategies of this newly emerged disease.

## Figures and Tables

**Table 1:** Reaction conditions used in PCR amplification and sequencing.

Gene	PCR Primers (Forward/Reverse)	PCR: Thermal Cycles: (Annealing Temp. in Bold)
ITS	ITS1/ITS4	94 °C: 3 min, (94 °C: 30 s, **55 °C**: 30 s, 72 °C: 45 s) × 33 cycles, 72 °C: 10 min
*CAL*	CL-1C/CL-2C	95 °C: 3 min, (95 °C: 30 s, **55 °C**: 30 s, 72 °C: 30 s) × 35 cycles, 72 °C: 10 min
*ACT*	ACT-512F/ACT-783R	94 °C: 3 min, (94 °C: 30 s, **58 °C**: 30 s, 72 °C: 45 s) × 35 cycles, 72 °C: 10 min
*TUB2*	T1/Bt-2b	95 °C: 3 min, (95 °C: 30 s, **55 °C**: 30 s, 72 °C: 30 s) × 35 cycles, 72 °C: 10 min
*CHS-1*	CHS-79F/CHS-354R	94 °C: 3 min, (94 °C: 30 s, **58 °C**: 30 s, 72 °C: 45 s) × 35 cycles, 72 °C: 10 min
*ApMat*	AM-F/AM-R	94 °C: 3 min, (94 °C: 1 min, **55 °C**: 30 s, 72 °C: 1 min) × 35 cycles, 72 °C: 10 min
*GAPDH*	GD-F1/GD-R1	94 °C: 3 min, (94 °C: 30 s, **58 °C**: 30 s, 72 °C: 45 s) × 35 cycles, 72 °C: 10 min

**Table 2:** Growth rate of representative *Colletotrichum* isolates on CM, PDA, MM, and CZA media.

Media	Colony Growth (mm/d)		
	SHS 1-3	SHS 1-6	SHS 1-7
CM	16.41 ± 0.24 b *	15.78 ± 0.14 b	17.87 ± 0.18 b
PDA	17.33 ± 0.23 a	17.93 ± 0.12 a	15.24 ± 0.16 a
MM	5.48 ± 0.08 d	5.01 ± 0.05 d	6.59 ± 0.12 d
CZA	13.36 ± 0.17 c	12.51 ± 0.13 c	11.25 ± 0.21 c

Data were analyzed with SPSS Statistics 19.0 by one-way ANOVA, and means were compared using Duncan’s test at a significance level of *p* = 0.05. * Different letters indicate the significant difference at *p* = 0.05 level.

**Table 3:** Growth rates of representative *Colletotrichum* isolates on 15, 20, 25, 30, and 35 °C.

Temperature	Colony Growth (mm/d)		
	SHS 1-3	SHS 1-6	SHS 1-7
15 °C	8.48 ± 0.26 d*	9.92 ± 0.06 e	9.53 ± 0.07 e
20 °C	15.43 ± 0.40 a	14.17 ± 0.27 c	15.4 ± 0.29 c
25 °C	13.70 ± 0.15 b	18.09 ± 0.19 a	17.14 ± 0.19 a
30 °C	12.42 ± 0.30 c	16.05 ± 0.31 b	16.14 ± 0.07 b
35 °C	7.78 ± 0.27 c	12.15 ± 0.30 d	10.89 ± 0.23 d

Data were analyzed with SPSS Statistics 19.0 by one-way ANOVA, and means were compared using Duncan’s test at a significance level of *p* = 0.05. * Different letters indicate the significant difference at *p* = 0.05 level.

**Table 4:** The infection severities of representative *Colletotrichum siamense* isolates on leaves of *Viburnum odoratissimum* after seven days.

Isolate	Species	Lesion Length (mm)
SHS 1-3	*C. siamense*	3.97 ± 0.90 a *
SHS 1-6	*C. siamense*	4.06 ± 0.96 a
SHS 1-7	*C. siamense*	3.99 ± 1.04 a

Data were analyzed with SPSS Statistics 19.0 by one-way ANOVA, and means were compared using Duncan’s test at a significance level of *p* = 0.05. * The same letter indicates no significant difference at the *p* = 0.05 level.

**Table 5:** Inhibitory effect of different fungicides on mycelial growth of representative isolate SHS 1-3, SHS 1-6, and SHS 1-7.

Fungicide	EC50 Values (μg/mL)		
	SHS 1-3	SHS 1-6	SHS 1-7
Prochloraz	0.0033 ± 0.0003 f *	0.0047 ± 0.00029 e	0.0039 ± 0.00030 d
Tebuconazole	0.13 ± 0.0010 cd	0.086 ± 0.0016 d	0.10 ± 0.0013 c
Myclobutanil	0.52 ± 0.038 b	0.22 ± 0.053 b	0.57 ± 0.044 b
Pyraclostrobine	0.096 ± 0.022 de	0.072 ± 0.017 de	0.067 ± 0.0019 cd
Carbendazim	0.071± 0.0011 e	0.051 ± 0.0023 de	0.044 ± 0.0014 cd
Flusilazole	0.068 ± 0.038 e	0.052 ± 0.041de	0.12 ± 0.0036 c
Iprodione	15.15 ± 1.25 a	12.27 ± 1.44 a	14.05 ± 0.96 a
Difenoconazole	0.15 ± 0.018 c	0.13 ± 0.031 cd	0.10 ± 0.020 c
Imazalil	0.17 ± 0.022 c	0.14 ± 0.035 bc	0.14 ± 0.017 c

Data were analyzed with SPSS Statistics 19.0 by one-way ANOVA, and means were compared using Duncan’s test at a significance level of *p* = 0.05. * Different letters indicate the significant difference at the *p* = 0.05 level.

## Data Availability

All data generated or analyzed during this study are included in this article.

## Supplementary Materials

The following supporting information can be downloaded at: https://www.mdpi.com/article/10.3390/jof9090882/s1, Table S1. Host, Origin, and GenBank accession numbers of strains of Colletotrichum species used for phylogenetic analyses.
