# Supplementary material for: Colletotrichum siamense, a Novel Causal Agent of Viburnum odoratissimum Leaf Blotch and Its Sensitivity to Fungicides
Source: J Fungi (Basel). 2023 Aug 28;9(9):882. doi: 10.3390/jof9090882 (PMC10532865; doi:10.3390/jof9090882)
Supplement: Supplementary file 1 [file jof-09-00882-s001.zip › jof-2403110-supplementary.pdf]

Table S1. Host, Origin, and GenBank accession numbers of strains of *Colletotrichum* species used for phylogenetic analyses.

Species	Isolates[1]	Host	Origin	GenBank accession number						
				ITS	*ACT*	*CAL*	*TUB2*	*CHS-1*	*ApMat*	*GAPDH*
C. aeschynomenes	ICMP 17673[T]	*Aeschynome-ne virginica*	USA	JX010176	JX009483	JX009721	JX010392	JX009799	KM360145	JX009930
C. alatae	CBS 304.67[T]	*Dioscorea alata*	India	JX010190	JX009471	JX009738	JX010383	JX009837	-	JX009990
C. alienum	ICMP 12071[T]	*Malus domestica*	New Zealand	JX010251	JX009572	JX009654	JX010411	JX009882	KM360144	JX010028
C. alienum	ICMP 18691	*Persea americana*	Australia	JX010217	JX009580	JX009664	JX010385	JX009754	-	JX010018
C. analogum	YMF1.06943	*Ageratina adenophora*	China	OK030860	OK513599	-	OK513629	OK513559	-	OK513663
C. asianum	ICMP 18580[T]	*Coffea arabica*	Thailand	FJ972612	JX009584	FJ917506	JX010406	JX009867	-	JX010053
C. asianum	ICMP 18696	*Mangifera indica*	Australia	JX010192	JX009576	JX009723	JX010384	JX009753	-	JX009915
C. camelliae	LC1364[T]	*Camellia sinensis*	China	KJ955081	KJ954363	KJ954634	KJ955230	-	KJ954497	KJ954782
C. clidemiae	ICMP 18658[T]	*Clidemia hirta*	USA, Hawaii	JX010265	JX009537	JX009645	JX010438	JX009877	KC888929	JX009989
C. dimorphum	YMF1.07303	*Ageratina adenophora*	China	OK030866	OK513605	-	OK513635	OK513565	-	OK513669
C. fructicola	ICMP 18581[T]	*Coffea arabica*	Thailand	JX010165	FJ907426	FJ917508	JX010405	JX009866	-	JX010033
C. fructicola	ICMP 18613	*Limonium sinuatum*	Israel	JX010167	JX009675	JX009491	JX010388	JX009772	-	JX009998
C. gloeosporioides	ICMP 17821[T]	*Citrus sinensis*	Italy	JX010152	JX009531	JX009731	JX010445	JX009818	-	JX010056

C. gracile	YMF1.06939	*Ageratina adenophora*	China	OK030868	OK513607	-	OK513637	OK513567	-	OK513671
C. helleniense	CBS 142418^{T}	*Poncirus trifoliata*	Greece, Arta	KY856446	KY856019	KY856099	KY856528	KY856186	-	KY856270
C. horii	ICMP 10492^{T}	*Diospyros kaki*	Japan	GQ329690	JX009438	JX009604	JX010450	JX009752	JQ807840	GQ329681
C. jiangxiense	LF687^{T}	*Camellia sinensis*	China	KJ955201	KJ954471	KJ954752	KJ955348	-	KJ954607	KJ954902
C. kahawae	ICMP 18539^{T}	*Olea europaea*	Australia	JX010230	JX009523	JX009635	JX010434	JX00980	-	JX009966
C. mengyinense	SAUCC200702^{T}	*Rosa chinensis*	China	MW786742	MW883695	MW922538	MW888970	MW883686	-	MW846240
C. mengyinense	SAUCC200913	*Juglans regia*	China	MW786690	MW883697	MW922540	MW888972	MW883688	-	MW876473
C. musae	ICMP 19119^{T}	*Musa* sp.	USA	JX010146	JX009433	JX009742	HQ596280	JX009896	KC888926	JX010050
C. musae	CBS 192.31	*Musa* sp.	Indonesia	JX010143	JX009587	JX009690	-	JX009841	-	JX009929
C. nanhuaense	YMF1.04990	*Ageratina adenophora*	China	OK030871	OK513610	-	OK513640	OK513570	-	OK513674
C. nullisetosum	YMF1.06946	mango	China	OK030872	OK513611	-	OK513641	OK513571	-	OK513675
C. nupharicola	ICMP 18187^{T}	*Nuphar lutea* subsp. *poly-sepala*	USA	JX010187	JX009437	JX009663	-	JX009835	-	JX009972
C. nupharicola	ICMP 17938			JX010189	JX009486	JX009661	JX010397	JX009834	-	JX009936
C. oblongisporum	YMF1.06938	*Ageratina adenophora*	China	OK030874	-	-	OK513643	OK513573	-	OK513677
C. psidii	ICMP 19120^{T}	*Psidium* sp.	Italy	JX010219	JX009515	JX009743	JX010443	JX009901	KC888931	JX009967
C. queenslandicum	ICMP 1778^{T}	*Carica papaya*	Australia	JX010276	JX009447	JX009691	JX010414	JX009899	KC888928	JX009934
C. queenslandicum	ICMP 1780	*Carica* sp.	Australia	JX010186	JX009504	JX009693	-	JX009900	-	JX010010

C. salsolae	ICMP 19051^{T}	*Salsola tragus*	Hungary	JX010242	JX009562	JX009696	JX010403	JX009863	KC888925	JX009916
C. salsolae	CBS 119296	*Glycine max*	Hungary	JX010241	JX009559	JX009695	-	JX009791	-	JX009917
C. siamense	CBS 130417	*Coffea arabica*	Thailand	JX010171	FJ907423	FJ917505	JX010404	JX009865	KP703769	JX009924
C. siamense	CBS 125378	*Hymenocallis americana*	China	JX010278	GQ856775	JX009709	JX010410	GQ856730	-	JX010019
C. siamense	**SHS 1-1**	***Viburnum odoratissimum***	**China**	**OQ848032**	**OQ858333**	**OQ858340**	**OQ858361**	**OQ858347**	**OQ858368**	**OQ858354**
	**SHS 1-2**			**OQ848033**	**OQ858334**	**OQ858341**	**OQ858362**	**OQ858348**	**OQ858369**	**OQ858355**
	**SHS 1-3**			**OQ135320**	**OQ148592**	**OQ148595**	**OQ148604**	**OQ148598**	**OQ858370**	**OQ148601**
	**SHS 1-4**			**OQ848034**	**OQ858335**	**OQ858342**	**OQ858363**	**OQ858349**	**OQ858371**	**OQ858356**
	**SHS 1-5**			**OQ848035**	**OQ858336**	**OQ858343**	**OQ858364**	**OQ858350**	**OQ858372**	**OQ858357**
	**SHS 1-6**			**OQ135323**	**OQ148593**	**OQ148596**	**OQ148605**	**OQ148599**	**OQ858373**	**OQ148602**
	**SHS 1-7**			**OQ135322**	**OQ148594**	**OQ148597**	**OQ148606**	**OQ148600**	**OQ858374**	**OQ148603**
	**SHS 1-8**			**OQ848036**	**OQ858337**	**OQ858344**	**OQ858365**	**OQ858351**	**OQ858375**	**OQ858358**
	**SHS 1-9**			**OQ848037**	**OQ858338**	**OQ858345**	**OQ858366**	**OQ858352**	**OQ858376**	**OQ858359**
	**SHS 1-10**			**OQ848038**	**OQ858339**	**OQ858346**	**OQ858367**	**OQ858353**	**OQ858377**	**OQ858360**
C. siamense	ICMP 18578^{T}	*Coffea arabica*	Thailand	JX010171	JX009518	JX009714	JX010404	JX009865	-	JX009924
C. temperatum	Coll883^{T}= CBS133122	*Vaccinium macrocarpon*	USA	JX145159	-	-	JX145211	MZ799254	JX145298	-
C. tropicale	ICMP 18653^{T}	*Theobroma cacao*	Panama	JX010264	JX009489	JX009719	JX010407	JX009870	-	JX010007
C. wuxiense	JS1A32^{T}= CGMCC 3.17894	*Camellia sinensis*	China	KU251591	KU251672	-	KU252200	KU251939	KU251722	KU252045
C. yunajiangence	YMF1.04996	*Ageratina adenophora*	China	OK030885	OK513620	-	OK513649	OK513583	-	OK513686

Colletotrichum boninense	CBS 123755	*Crinum asiaticum* var. *sinicum*	Japan	JQ005153	JQ005501	JQ005674	JQ005588	JQ005327	-	JQ005240

Strains isolated from the current study are in bold. ^{T} = Ex-type culture.

[1] *ICMP*: International Collection of Microorganisms from Plants, Auckland, New Zealand; *CBS*: Culture collection of the Westerdijk Fungal Biodiversity Institute, Utrecht, The Netherlands; *YMF*: Herbarium of the Laboratory for Conservation and Utilization of Bio-resources, Yunnan University, Yunnan, China; *LF*: Working collection of Fang Liu, housed at CAS, China; *LC*: Working collection of Lei Cai, housed at CAS, China; *SAUCC*: Shandong Agricultural University Culture Collection,China; *CGMCC*: China General Microbiological Culture Collection Center, Beijing, China.
